# Medial Malleolar Fracture Fixation with Stainless Steel, Titanium, Magnesium, and PLGA Screws: A Finite Element Analysis

**DOI:** 10.3390/jfb17020059

**Published:** 2026-01-24

**Authors:** Mehmet Melih Asoglu, Volkan Kızılkaya, Ali Levent, Huseyin Kursat Celik, Ozkan Kose, Allan E. W. Rennie

**Affiliations:** 1 Orthopedics and Traumatology Clinic, Private Metrolife Hospital, Sanliurfa 63320, Turkey; melihasoglu@hotmail.com (M.M.A.); dr_volkankizilkaya@hotmail.com (V.K.); mdalilevent@yahoo.com (A.L.); 2 Department of Agricultural Machinery and Technology Engineering, Akdeniz University, Antalya 07070, Turkey; hkcelik@akdeniz.edu.tr; 3 Engineering Department, Lancaster University, Lancaster LA1 4YW, UK; a.rennie@lancaster.ac.uk; 4 Department of Orthopedics and Traumatology, Antalya Training and Research Hospital, University of Health Sciences, Antalya 07100, Turkey

**Keywords:** medial malleolus, ankle fracture, finite element analysis, titanium, stainless steel, magnesium, PLGA, micromotion, von Mises stress, biodegradable implants

## Abstract

Background: Implant material may influence interfragmentary mechanics in medial malleolar (MM) fracture fixation. This study aimed to compare stainless steel, titanium, magnesium, and PLGA screws under identical conditions using finite element analysis (FEA). Methods: A CT-based ankle model with a unilateral oblique MM fracture (θ = 60° to the medial tibial plafond) was fixed with two parallel M4 × 35 mm screws placed perpendicular to the fracture plane (inter-axial distance 13 mm). Contacts were defined as nonlinear frictional, and each screw was assigned a pretension force of 2.5 N. Static single-leg stance was simulated with physiologic tibia/fibula load sharing. Four scenarios differed only by screw material. Primary outputs were interfragmentary micromotion (maximum sliding and gap). Secondary measures included fracture interface contact/frictional stresses, screw/bone von Mises stress, global construct displacement, and average tibiotalar cartilage contact pressure. Results: Interfragmentary micromotion increased as screw stiffness decreased. Maximum sliding was 32.2–33.8 µm with stainless steel/titanium, 40.4 µm with magnesium, and 65.0 µm with PLGA; corresponding gaps were 31.2–32.0 µm with stainless steel and titanium, 31.2 µm with magnesium, and 54.1 µm with PLGA, respectively. Interface stresses followed the same pattern: contact pressure (3.18–3.24 MPa for stainless steel/titanium/magnesium vs. 4.29 MPa for PLGA); frictional stress (1.46–1.49 MPa vs. 1.98 MPa). Peak screw von Mises stress was highest in stainless steel (104.1 MPa), then titanium (73.4 MPa), magnesium (47.4 MPa), and PLGA (17.9 MPa). Global axial displacement (0.26–0.27 mm) and average tibiotalar cartilage contact pressure (0.73–0.75 MPa) were essentially unchanged across materials. All conditions remained below commonly cited thresholds for primary bone healing (gap < 100 µm); however, PLGA exhibited a reduced safety margin. Conclusions: Under identical geometry and loading conditions, titanium and stainless steel yielded the most favorable interfragmentary mechanics for oblique MM fixation; magnesium showed intermediate performane, and PLGA produced substantially greater micromotion and interface stresses. These findings support the use of metallic screws when maximal initial stability is required and suggest that magnesium may be a selective alternative when reducing secondary implant removal is prioritized.

## 1. Introduction

Ankle fractures are among the most prevalent skeletal injuries. In a recent Swedish registry-based analysis encompassing 27,169 fractures, ankle fractures accounted for 10.3% of all adult fractures [1]. Anatomically, ankle fractures may involve the medial, lateral, or posterior malleolus, either in isolation or in combination. Population-based data indicate that nearly 1 in 4 ankle fractures involve the medial malleolus (MM) [2,3]. Stable, minimally displaced (<2 mm) isolated medial malleolar fractures can be managed non-operatively [4]. However, unstable configurations, such as displaced MM fractures and bimalleolar or trimalleolar injuries, generally require surgical fixation to restore mortise congruity and avert increases in cartilage contact pressures, and reduce the risk of post-traumatic osteoarthritis [5,6,7]. Current treatment goals are anatomic reduction, maintenance of talar position, and sufficiently stable fixation to support early range of motion and, when appropriate, progressive weight-bearing [5,8]. A variety of fixation strategies is used depending on fracture geometry, fragment size, bone quality, and soft-tissue status [9]. For distal, small-fragment injuries, Kirschner wires with tension-band wiring can be considered [10]. Transverse/oblique MM fractures are most commonly fixed with two partially threaded cancellous lag screws inserted perpendicular to the fracture plane. In contrast, vertical shear fractures often require lag screws with a buttress plate to prevent superior migration [9,11]. The choice of implant and configuration is therefore tailored to the fracture pattern and biology.

Despite successful union, hardware prominence and soft-tissue irritation frequently lead to secondary implant removal after ankle fracture fixation, increasing morbidity and healthcare cost [12,13,14]. In a nationwide Finnish registry study of 83,666 operatively treated ankle fractures, Happonen et al. found that implant removal was the most common long-term reoperation, occurring in 11% at 1 year and 17% at 3 years [15]. Nevertheless, implant removal is not a benign procedure and is associated with a meaningful complication burden, with reported adverse event rates as high as 14% for removal procedures [12]. These considerations have intensified interest in strategies that reduce the need for planned or symptomatic hardware removal.

Biodegradable implants, particularly magnesium (Mg) alloys and polymer systems such as polylactic-co-glycolic acid (PLGA), may offer theoretical advantages by gradually resorbing, thereby eliminating the need for routine removal and reducing long-term imaging artifacts [16,17]. Early clinical reports in ankle and foot surgery suggest that both Mg-based and polymer screws can achieve union with acceptable functional outcomes and, in some series, show lower removal rates than titanium screws [18,19,20,21]. However, the mechanical performance of resorbable implants in high-load environments remains a central concern. Computational and experimental data consistently show that titanium exhibits the highest strength and the least deformation under physiologic and worst-case loading; magnesium alloys demonstrate intermediate performance; and polymers perform worst, with notable greater displacement under load [22,23,24]. In addition, bioabsorbable materials lose mechanical strength over time in vivo, making fixation durability sensitive to the pace of fracture healing. Each biodegradable class has specific limitations. Magnesium’s relatively rapid corrosion can generate hydrogen gas and radiolucent zones; although usually transient, these phenomena may complicate radiographic interpretation and, in rare cases, local biology [18,19,20,25]. Polymer screws generally provide lower initial strength and are more susceptible to creep and deformation under sustained loading. Several models and in-vitro studies report inferior mechanical stability compared with metallic screws, making careful case selection essential [21,22,23].

Conventional metallic implants (stainless steel and titanium) remain the workhorse for MM fracture fixation, but they entail substantial secondary hardware removal. Biodegradable alternatives could mitigate this burden, provided their initial mechanical behavior is adequate for the MM fracture environment [16]. Therefore, this finite element study investigated whether screw material (Ti-6Al-4V, 316LVM stainless steel, MgY4RE3Zr, and PLGA) affects interfragmentary micromotion (sliding and gap) in a 60° oblique MM fracture fixed with two parallel lag screws under static single-leg stance loading, and how material selection influences stress distribution within the screws and at the fracture interface. We hypothesized that screw material would significantly influence construct stability, with metallic screws producing the lowest interfragmentary micromotion, magnesium demonstrating intermediate behavior, and PLGA resulting in the highest micromotion under the same loading condition.

## 2. Materials and Methods

### 2.1. Study Design and Overview

We conducted a finite element analysis (FEA) under a static stance loading assumption to compare the biomechanical performance of four different screw materials: stainless steel (18Cr-14Ni-2.5Mo), titanium alloy (Ti-6Al-4V), magnesium alloy (MgY4RE3Zr), and polymer (PLGA), for the fixation of a typical medial malleolar fracture. Anatomy, fracture geometry, screw size/position, loading, contacts, and meshing strategy were kept identical across scenarios. An identical fixation configuration consisting of two parallel malleolar screws inserted perpendicular to the fracture plane was analyzed under similar conditions in four separate simulations, each employing a different screw material (Table 1).

### 2.2. Modeling of the Medial Malleolar Fracture

**Figure 1:**
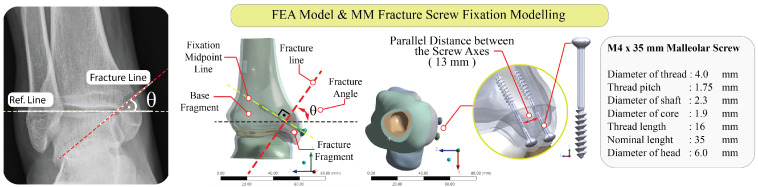
Model setup and fixation parameters for an oblique medial malleolar fracture.

Computerized tomography (CT)-derived anatomy from a single healthy adult male (height 184 cm, weight 98 kg) was used to build the intact ankle model. Imaging was obtained in the emergency department due to a suspected ankle fracture, but no osseous pathology or congenital/acquired deformity was identified on review. Scans were acquired supine at 130 kV/42 mA with 0.7-mm slices, covering from 80 mm proximal to the ankle joint to the heel, yielding 334 axial images. The ankle model was generated and segmented into cortical bone, trabecular bone, and articular cartilage layers to reproduce realistic thickness distributions and bone–cartilage contact areas for the tibia and talus. Segmentation and solid modelling were performed using 3D Slicer (version 5.8.1), Meshmixer (version 3.5.0), and SolidWorks (version SP5), while the loading simulations were carried out in the Structural Mechanics Module of ANSYS Workbench (ANSYS Workbench 2025. R1.), a commercial FEA code. Because routine CT does not delineate articular cartilage with sufficient contrast for direct segmentation, the cartilage layers were generated using a geometry-based approach. Briefly, tibial and talar bone surfaces were segmented from CT in 3D Slicer, and cartilage was then constructed by offsetting the corresponding subchondral bone surfaces to create continuous articular layers, followed by smoothing and trimming to the native tibiotalar contact region using Meshmixer and SolidWorks prior to meshing in ANSYS Workbench. An isolated oblique MM fracture was created on the distal tibia; the fracture angle θ was set to 60° relative to the reference line on the medial tibial plafond. Fixation was achieved with two parallel malleolar screws (M4 × 35 mm) placed perpendicular to the fracture plane. Screw axes were kept parallel with a 13 mm inter-axial distance. Screw threads were modeled using the original buttress geometry (pitch 1.75 mm, leading/trailing flank angles 45°/7°) to capture realistic bone engagement (Figure 1).

### 2.3. Material Properties

Material constants for cortical bone, trabecular bone, and articular cartilage were taken from studies commonly adopted in validated ankle FE models in the related literature (Table 2) [26,27,28,29,30,31,32,33,34]. Metallic screw materials in the FEA were modelled as a bilinear isotropic hardening material model to evaluate the potential for realistic permanent plastic deformation. By contrast, PLGA screws were represented under an isotropic, homogeneous linear elastic assumption, as PLGA is an amorphous copolymer lacking a crystalline lattice and therefore exhibits no pronounced anisotropy at the macroscopic scale (Table 3) [35,36]. In accordance with anatomical reality, trabecular and cortical bones were modelled as bonded to represent their natural continuity. In contrast, all other interfaces were modeled as nonlinear frictional contacts to achieve a realistic representation of the fixation construct

### 2.4. Contacts and Constraints

Nonlinear surface-to-surface frictional contact was assigned at all interfaces where relative motion may occur. These interfaces included the fracture apposition surfaces, the screw–bone interfaces, and the tibiotalar joint cartilaginous articulation. Bonded contact was assigned between cortical and trabecular bone and between subchondral bone and cartilage to prevent separation and sliding. The friction coefficients used for cartilage–cartilage, bone–screw, and bone–bone interfaces were identical across scenarios and are presented in Table 4 [37,38,39,40,41]. To reproduce the clamping force generated by tightening, each screw was subjected to an axial pretension of 2.5 N before external loading [41]. The pretension was applied to establish a stable initial contact state between the screw threads and bone before external loading. Therefore, a relatively low and uniform pretension value was selected to ensure consistent initial engagement across simulations and to avoid material-dependent bias from torque–preload relationships that were not explicitly modeled [41]. These contact formulations, friction values, and preload magnitudes were identical in all simulations to isolate the effect of screw material.

### 2.5. Boundary Conditions

**Figure 2:**
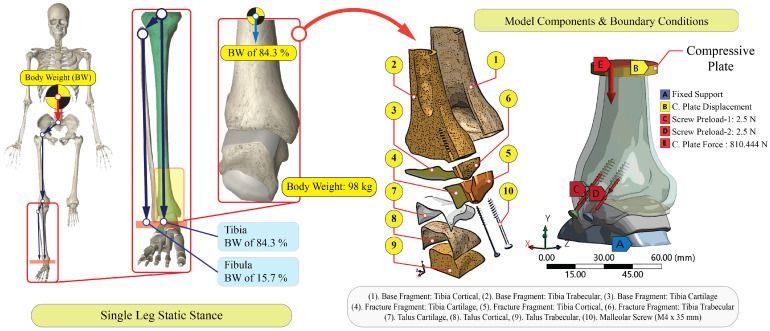
Boundary conditions and model components.

To simulate a single-leg static stance in neutral, a superior compressive plate applied an axial load equivalent to the subject’s body weight (98 kg). Consistent with classic load-sharing data, 84.3% of the axial load was assigned to the tibial column and 15.7% to the fibular column; thus, the tibia experienced 810.444 N (98 kg × 9.81 m/s^2^ × 0.843) [42]. The compressive plate acted along the Y-axis (proximal-to-distal direction). The proximal tibial shaft was constrained to eliminate rigid-body motion while allowing axial compression; the defined contacts governed distal kinematics. The talus was supported on an inferior foundation that transmitted contact forces while preventing gross translation and rotation of the ankle–foot complex. No additional external moments were applied. All other degrees of freedom were free unless restricted by contact formulations. All loads, constraints, and result components were defined and evaluated within a fixed global Cartesian coordinate system, where the Y-axis represents the proximal–distal direction of the tibia, the X-axis the medial–lateral direction, and the Z-axis the anterior–posterior direction; this coordinate system was retained unchanged across all simulations. The boundary conditions are illustrated in Figure 2. The fixed support, compressive plate loading, and screw pretension locations were uniformly applied across all tested scenarios.

### 2.6. FE Model Mesh Structure

**Figure 3:**
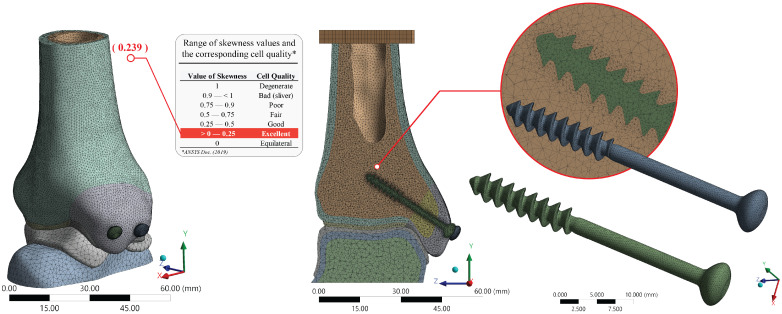
Meshing details.

A three-dimensional finite element model of the tibia–talus complex with malleolar screw fixation was generated and meshed using a curvature-based approach in ANSYS Workbench. The same meshed geometry was consistently employed across four analyses, differing only in the screw material definition, to ensure methodological comparability. To ensure the discretization’s validity, mesh quality was assessed using the skewness metric, which confirmed that the elements fell within the range typically classified as good to excellent according to ANSYS verification standards [43]. This provided confidence that the mesh resolution and element quality were sufficient to capture the mechanical response of the fixation construct under the prescribed loading conditions [44]. A summary of meshing details with quality metrics is shown in Figure 3. A separate mesh refinement–based convergence analysis was not performed; instead, numerical robustness was ensured by employing a skewness element metric check, as mentioned previously, a high-order discretization, and retaining the same mesh across all simulations. Under this framework, the use of Tet10, Hex20, and Wed15 elements (893,150 elements and 1,330,577 nodes) allowed for consistent and reliable comparisons across material scenarios, as solution differences are not influenced by mesh refinement effects (Table 5).

### 2.7. Outcome Measures

**Figure 4:**
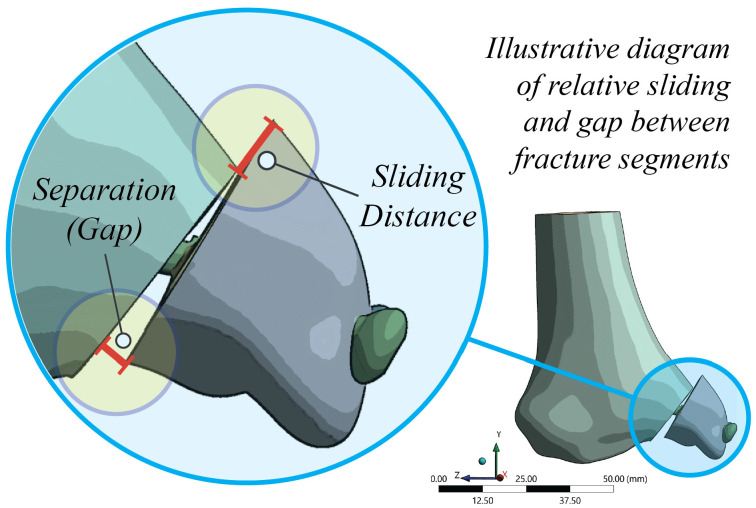
Definition of micromotion metrics at the fracture plane. Schematic showing how sliding distance (tangential motion) and separation/gap (opening) were obtained between paired points on the opposing fracture surfaces along the fixation line; peak values at the end of the loading step were used for analysis.

The primary outcome measures quantified construct integrity under maximum stance loading. Fragment micromotion at the fracture plane was assessed in terms of tangential relative displacement (“sliding distance”) and normal opening displacement (“separation” or “gap”), obtained directly as analysis outputs describing the relative motion between assembled solid model components across the opposing fracture surfaces. The maximum sliding and gap values at the fracture interface, as determined by the solution process, were recorded. In addition, von Mises stress distributions were extracted for the fixation screws and the adjacent bone, and the maximum values were reported, together with their corresponding stress maps. Global construct displacement was simultaneously quantified as the resultant motion of the medial malleolar fragment. The definitions of sliding and gap measurements are schematically illustrated in Figure 4.

### 2.8. Assumptions on Interfragmentary Micromotion and Fracture Healing

Previous studies have shown that if the gap at the fracture interface is less than 100 µ and the interfragmentary strain is less than 2%, the fracture unites through primary bone healing. Accordingly, a displacement threshold of 100 µm was adopted as the upper limit for primary fracture healing in this study [45,46,47].

## 3. Results

**Figure 5:**
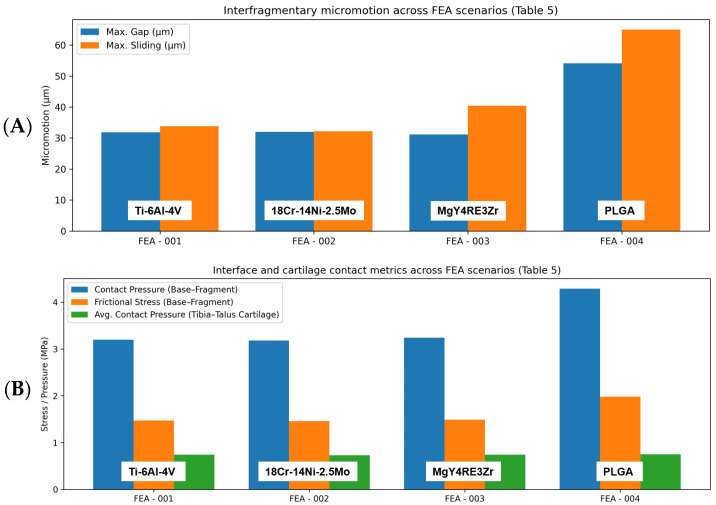
Finite element (FEA) outcome metrics across screw material scenarios. (**A**) Interfragmentary micromotion at the medial malleolus fracture interface under the peak stance loading condition, reported as maximum separation (gap) and maximum sliding distance (µm). (**B**) Interface and joint contact measures for the same loading condition, including contact pressure and frictional stress between the tibial base and fracture fragment (MPa), and the average contact pressure on the tibiotalar cartilage surface (MPa). FEA-001: Ti-6Al-4V; FEA-002: 18Cr-14Ni-2.5Mo stainless steel; FEA-003: MgY4RE3Zr; FEA-004: PLGA.

Four finite element scenarios were analyzed under identical simulation conditions. Micromotion at the fracture plane increased as screw stiffness decreased. Maximum sliding distance increased from 32.2–33.8 µm with titanium and stainless steel to 40.4 µm with magnesium and 64.97 µm with PLGA. The corresponding gap (separation) increased from 31.8–31.9 µm (Titanium/Stainless Steel) to 31.1 µm (Mg) and 54.08 µm (PLGA). Interface mechanics reflected the same trend. Contact pressure and frictional stress between fracture fragments were clustered at 3.18–3.24 MPa and 1.46–1.49 MPa for Ti/SS/Mg, but increased to 4.29 MPa and 1.98 MPa with PLGA. Consistent with this, the average tibiotalar cartilage contact pressure on the base surface remained essentially unchanged across materials (0.73–0.75 MPa) (Table 6) (Figure 5).

**Figure 6:**
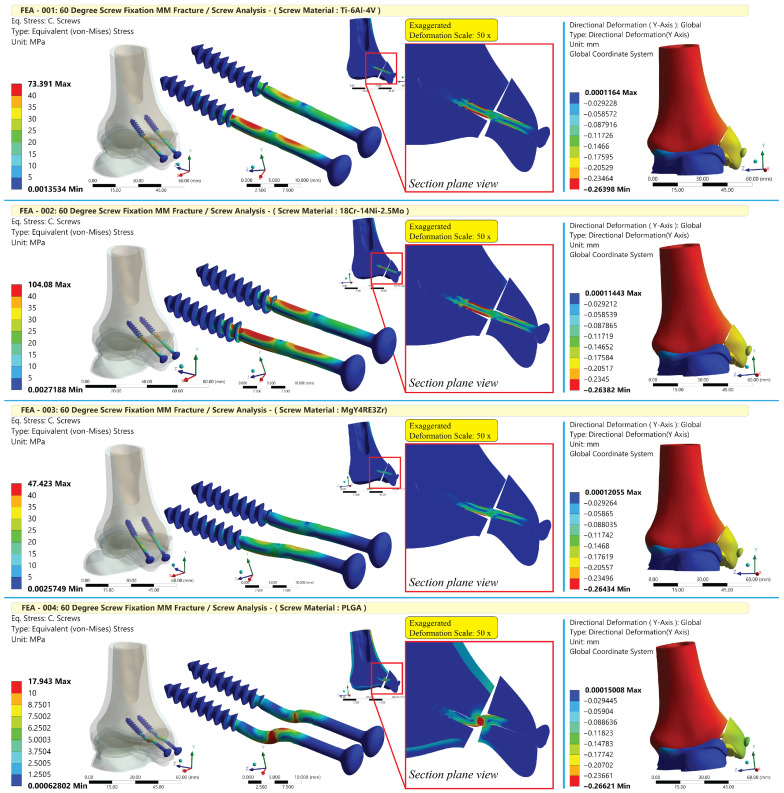
Visual output of the FEA scenarios.

Material substitution redistributed stresses within the construct. Peak von Mises stress in the screws was highest for stainless steel (104.10 MPa), followed by titanium (73.39 MPa), magnesium (47.42 MPa), and PLGA (17.94 MPa). Despite these material-dependent differences in local measures, the global axial displacement of the construct (Y-axis) was nearly identical among scenarios (0.26–0.27 mm). Overall, titanium and stainless steel exhibited the lowest micromotion and the lowest interface stresses. In comparison, PLGA produced substantially greater sliding and separation and higher contact/frictional stresses at the fracture, with magnesium showing intermediate behavior. These findings are visualized in the result graphs (Figure 6) and summarized in Table 7.

## 4. Discussion

This study evaluated the biomechanical effects of screw material in oblique medial malleolar fractures fixed with two parallel lag screws using finite element analysis. Across otherwise identical constructs, screw material substantially influenced interfragmentary mechanics under peak stance loading. Stainless steel and titanium produced the lowest micromotion (maximum sliding 32–34 µm; gap 32 µm), magnesium was intermediate (sliding 40 µm; gap 31 µm), and PLGA generated the greatest micromotion (sliding 65 µm; gap 54 µm). Although these values remained below commonly cited thresholds for primary bone healing (<100 µm gap, <2% interfragmentary strain) [45,46,47], the relative increase with PLGA indicates a narrower biomechanical safety margin under the modeled loading. Material substitution also redistributed internal stresses: the peak von Mises stress in the screws decreased with decreasing stiffness (highest in stainless steel, lowest in PLGA), while contact and frictional stresses at the fracture interface were highest with PLGA, consistent with its greater relative motion. Notably, global construct displacement and mean tibiotalar cartilage contact pressure were essentially unchanged across materials, underscoring that the clinically salient differences lie in the fracture–implant microenvironment rather than in joint-level mechanics during static stance.

Our ranking of stability (Ti/SS > Mg > PLGA) aligns with computational and experimental studies showing that metallic implants provide greater initial stiffness and lower micromotion than bioresorbables, with magnesium occupying an intermediate position [48,49]. Comparable material sensitivity has been documented in other lower-limb constructs. For example, finite element models of tibial tubercle osteotomy and femoral neck fixation show greater fragment displacement and altered stress mapping when magnesium or polymeric screws are substituted for titanium, especially under higher loads [24,50]. Clinical data from ankle and foot surgery broadly fit this mechanical gradient. Early clinical series and mid-term cohorts report reliable union and good function with magnesium screws for MM fractures and chevron-type osteotomies, together with characteristic but transient radiographic radiolucent zones or gas that rarely compromise healing [19,20,51,52]. Comparative reports on biplanar chevron osteotomy and medial malleolar fractures suggest that magnesium is clinically non-inferior to titanium, with no implant removals [18,19,20]. Collectively, these data suggest a trade-off between maximizing early stability and minimizing secondary hardware removal, rather than a simple hierarchy of “better” and “worse” materials [51,52]. In pediatric patients, multicentre cohorts using PLGA screws have reported clinical outcomes and complication rates comparable to those with metal screws, with a reduced need for reoperation due to implant removal [53]. Contemporary series of osteochondral fracture fixation with PLGA likewise emphasize the value of avoiding secondary procedures, while calling for larger confirmatory studies [54,55,56]. However, in vitro and FEA work consistently demonstrate higher displacement and creep propensity in polymers under load, supporting careful indication selection, particularly in high-demand settings [49]. Overall, the clinical literature suggests that polymers can succeed when reduction and compression are excellent, and loading is moderated. Still, they are more vulnerable to instability under aggressive early loading [49,53,54,55,56].

The observed hierarchy reflected the materials’ elastic modulus and time-dependent behavior. Titanium and stainless steel maintain a low-motion, high-constraint regime at the fracture plane, limiting interfragmentary sliding and gap formation. Magnesium, with lower stiffness and strength, permits modestly greater sliding while redistributing stress away from the screw; explant-based mechanical testing combined with modeling shows a decline in bending strength over the first 6–12 weeks, consistent with progressive corrosion and evolving load sharing with bone [48]. PLGA undergoes hydrolytic degradation with stepwise decreases in molecular weight and strength over weeks to months; geometric changes can create partial self-locking, while acidic by-products transiently lower local pH. In HA-reinforced PLLA systems, FEA demonstrates deformation below approximately 0.15 mm under moderate loads, suggesting adequacy for primary callus formation, whereas unreinforced polymers deform more under identical loading [49]. Our numeric pattern is therefore consistent with modulus-driven micromotion: decreasing screw stiffness reduces peak implant stress but increases interface motion and frictional stresses. Similar material-dependent behavior has been reported in FE studies of Fulkerson tibial tubercle osteotomy and femoral neck fixation, where substituting magnesium or polymer screws for titanium increased fragment displacement and altered local stress distributions [24,50]. In the present model, sub-100 µm gaps across all materials anchor interpretation in biologically meaningful territory widely used in fracture-healing mechanics, while the relative differences clarify how much safety margin is lost when moving from metal to degradable systems [45,46,47,57,58].

Clinically, these trade-offs translate into material-specific indication windows. In cases of vertically oriented, comminuted, or osteoporotic MM patterns, where maximal initial stability is paramount, and micromotion tolerance is minimal, titanium or stainless steel remains the most conservative option [59,60]. Magnesium appears to be a rational choice for well-reduced fractures with robust lag compression, provided that eliminating planned hardware removal is a significant priority. In such cases, surgeons must be prepared to anticipate and recognize predictable radiographic changes associated with corrosion. It is the responsibility of surgeons to provide patients with the necessary counsel and guidance regarding these changes [19,20,51,52]. Conversely, our findings suggest caution in the use of standalone PLGA screws for high-demand adult MM fixation, particularly without adjuncts. Polymer screws offer patient-centered advantages, including the absence of a removal procedure, excellent MRI compatibility, and potential tissue friendliness near physes [61]. However, lower initial rigidity and greater creep propensity necessitate buttressing stability through meticulous reduction, optimized screw trajectory and pretension, more protective rehabilitation, and, where appropriate, hybrid or augmented constructs. In polymer-based fixations, rehabilitation should be deliberately staged to ensure that interface motion remains within sub-threshold ranges (approximately <0.10–0.15 mm) during the early healing window [49]. A synthesis of clinical observations aligns with the findings of our finite element analysis (FEA). Metals exhibit the most robust coupling at the fracture plane, magnesium modulates implant stress and removal rates while exhibiting slightly elevated micromotion, and polymers necessitate compensation strategies to attain equivalent stability during early loading. This underscores the importance of considering case-specific priorities, such as prioritizing early stability or minimizing planned hardware removal, in selecting the optimal material for a given procedure [20,51,52]. The near-constancy of mean tibiotalar contact pressure and global construct displacement across materials explains why some clinical series report similar functional scores despite different reoperation rates: material choice predominantly influences the biology at the fracture interface rather than average joint pressures during neutral static stance. This is particularly relevant for polymers, where careful, gradual loading may compensate for lower initial rigidity by keeping micromotion below biologically critical thresholds [62].

Key strengths of this study include a tightly controlled head-to-head design that isolates the material variable while holding constant the anatomy, fracture geometry (unilateral 60° oblique medial malleolus), screw size/trajectory, and standardized two-screw construct, contact definitions, meshing strategy, and loading conditions. This approach supports attributing between-material differences primarily to implant material rather than to confounding technical factors. Another strength is the use of clinically interpretable interface-level outcomes—interfragmentary sliding and gap—whose magnitudes map directly onto commonly accepted micromotion thresholds for primary bone healing [45,46,47,57,58]. The consistently sub–100 μm interfragmentary gaps across materials further anchor the inferences in established mechanobiological principles of direct (primary) fracture healing.

Several limitations should be acknowledged.

1. Model generalizability: This study represents a single CT-derived ankle, a single fracture pattern (60° oblique medial malleolus), and a single fixation strategy (two parallel lag screws). Different fracture morphologies (e.g., vertical shear, comminution), bone quality, screw trajectories, or augmented constructs (e.g., buttress plate, washers, hybrid constructs) may alter the relative performance of materials.
2. Loading conditions: Loading was limited to a static single-leg stance configuration. Real-world ankle mechanics include multiplanar forces (torsion/shear) and repetitive cyclic loading during gait; fatigue and high-demand events (e.g., stumble scenarios) were not modeled and could magnify material-dependent differences.
3. Material/contact assumptions: Homogeneous, isotropic, literature-based material properties and idealized (though standardized) friction/contact definitions and preload assumptions were used. These simplifications cannot fully capture patient-specific variability in anisotropy, bone density, thread purchase, and biological evolution during healing.
4. Time-dependent behavior not modeled: Only time-zero mechanics were evaluated. The progressive degradation/corrosion of magnesium and the hydrolytic degradation and creep of polymers—and the resulting changes in stiffness/strength over time—were not simulated.
5. Lack of experimental validation: As with all FEA studies, predictions are sensitive to constitutive and modeling assumptions. Although mesh quality metrics were within accepted ranges, benchtop testing and prospective clinical data are still needed to validate and translate these findings [48,49,50].

Future work should evaluate patient-specific variability (e.g., bone density and fragment size), incorporate multiplanar and cyclic gait-like loading (including stumble scenarios), and implement time-dependent degradation models for biodegradable implants with evolving material properties. Hybrid or augmented constructs should also be explored to balance early stability with the potential benefits of degradable materials. Clinically, prospective comparative studies stratified by fracture morphology, bone quality, and weight-bearing protocols are needed to confirm whether magnesium can reduce implant removal without compromising union, alignment, or function, and to define safe indications for polymer implants in adult versus pediatric ankles.

## 5. Conclusions

In conclusion, under identical geometry and loading, titanium and stainless steel provided the most favorable interfragmentary mechanical environment for oblique medial malleolus fixation, magnesium demonstrated intermediate behavior, and PLGA generated the greatest micromotion and interface stresses. Although all materials remained within ranges generally considered compatible with primary bone healing, the reduced safety margin observed with PLGA suggests caution in high-demand settings or when early rigid stability is critical. These findings support continued use of metallic screws when maximal initial stability is the priority and suggest that magnesium may represent a pragmatic alternative in carefully selected cases where minimizing secondary hardware removal is a key clinical objective. In contrast, polymer screws should be reserved for indications in which their patient-centered benefits outweigh lower initial rigidity and are paired with meticulous technique and appropriately staged rehabilitation. Importantly, these comparisons reflect time-zero, static behavior only; time-dependent phenomena such as polymer creep/viscoelasticity and biodegradation (including magnesium corrosion) were not modeled; therefore, the present results should not be interpreted as long-term in vivo performance.

## Figures and Tables

**Table 1:** Description of the FEA Scenarios.

Description of the FEA Scenarios	FEA Scenario Code	Screw Materials
2 × M4 × 35 mm—Parallel malleolar screw fixation	FEA-001	Ti-6Al-4V—(Titanium, alpha-beta alloy, cast)
	FEA-002	18Cr-14Ni-2.5Mo—(Eq. to—SS steel, austenitic, AISI 316LVM, cold worked)
	FEA-003	MgY4RE3Zr—(Magnesium Alloy, ASTM WE43A, cast)
	FEA-004	PLGA (unfilled), Polylactic-glycolic acid—bioabsorbable polymer

Abbreviations: AISI: American Iron and Steel Institute, ASTM: American Society for Testing and Materials, Ti-6Al-4V: Titanium alloy with 6% Aluminum and 4% Vanadium (alpha–beta alloy), 316LVM: Vacuum-melted low-carbon austenitic stainless steel, WE43A: Magnesium alloy containing Yttrium, Rare-Earth elements, and Zirconium, MgY4RE3Zr: Magnesium alloy with 4% Yttrium, 3% Rare-Earths, and Zirconium, PLGA: Polylactic-glycolic acid, M4: ISO metric screw thread.

**Table 2:** Material properties for the biological components.

Parameters	Cortical Bone	Trabecular Bone	Cartilage
Modulus of Elasticity (MPa)	19,100	1000.61	12
Yield Stress (MPa)	111	8.50	-
Ultimate Stress (MPa)	124	11.20	-
Poisson’s Ratio	0.30	0.30	0.42
Density (kg·m^−3^)	1980	830	431

**Table 3:** Material properties for the fixation screws.

Parameters	Fixation Screw (M4 × 35 mm)			
	Ti-6Al-4V *	18Cr-14Ni-2.5Mo *	MgY4RE3Zr *	PLGA **
Modulus of Elasticity (MPa)	113,800	187,500	44,600	1889
Yield Stress (MPa)	840	848	189.90	48
Ultimate Stress (MPa)	930	1034	252.50	48.20
Poisson’s Ratio	0.34	0.33	0.31	0.40
Density (kg·m^−3^)	4430	7990	1845	1348
Tangent Modulus (MPa)	1870	2000	1120	-

* Bilinear Isotropic Hardening, ** Isotropic elasticity.

**Table 4:** Coefficients of friction and fixation screw preload assigned in the FEA setup.

Parameters	Components in Relation	Value
Coefficient of Friction between	Cartilage and Cartilage	0.0164
	Bony Parts and Fixation Screw	0.37
	Bony Parts	0.46
Fixation Screw Preload (N)	2.5	

Abbreviations, N: Newton.

**Table 5:** Details of the FEA Models.

Meshing Approach	Curvature Based			
Average Skewness Value/Quality Measure	0.239 ± 0.01/Excellent			
Element Types (ANSYS WB Code)	Tet10/Hex20/Wed15			
Max. Element Size (mm)	6			
Min. Element Size (mm)	1 ^1^–2 ^2^			
Fixation Screw Contact Surface Size (mm)	0.30			
Defeature Size (mm)	0.03			
Curvature Min. Size (mm)	0.06			
Element Growth Rate	1.50			
Cortical Bone	# of Elements	259,317	# of Nodes	392,832
Trabecular Bone	# of Elements	495,796	# of Nodes	708,608
Cartilage	# of Elements	45,422	# of Nodes	25,821
Screws	# of Elements	110,452	# of Nodes	174,602
Complete Model (Includes Compressive Plate)	# of Elements	893,150	# of Nodes	1,330,577

^1^ Tibia Components, ^2^ Talus Components & Compressive Plate.

**Table 6:** FEA numerical results.

FEA Code	Max. Separation (Gap)	Max. Sliding Distance	Contact Pressure Between Base and Fracture Fragment	Frictional Stress Between Base and Fracture Fragment	Average Contact Pressure on Cartilage Surface Between Tibia and Talus (Base Surface)
	(µm)	(µm)	(MPa)	(MPa)	(MPa)
FEA-001	31.88	33.84	3.20	1.47	0.74
FEA-002	31.99	32.22	3.18	1.46	0.73
FEA-003	31.19	40.44	3.24	1.49	0.74
FEA-004	54.08 *	64.97 *	4.29 *	1.98 *	0.75 *

* Bold values indicate maximum values.

**Table 7:** Maximum equivalent (von Mises) stress by model components and maximum global displacement across FEA scenarios.

FEA Study Code	Max. Eq. Stress by Components						Max. Directional Displacement
	Tibia Cortical—Base Fragment	Tibia Cortical—Fracture Fragment	Tibia Trabecular—Base Fragment	Tibia Trabecular—Fracture Fragment	Tibia Cartilage—Base Fragment	Fixation Screws	Total (Y-Axis)
	(MPa)	(MPa)	(MPa)	(MPa)	(MPa)	(MPa)	(mm)
FEA-001	17,900	11,440	12,390	8720	0.749	73,390	0.264
FEA-002	17,960	13,110	11,570	7892	0.746	104,100	0.264
FEA-003	17,750	8958	14,360	11,260	0.754	47,420	0.264
FEA-004	16,840	5745	24,120	23,270	0.795	17,940	0.266

## Data Availability

The data supporting the findings of this study are available from the corresponding author upon reasonable request.

## Abbreviations

The following abbreviations are used in this manuscript:

AISI	American Iron and Steel Institute
ANSYS	ANSYS Workbench Structural Mechanics Module
ASTM	American Society for Testing and Materials
CT	Computerized tomography
FEA	Finite element analysis
MM	Medial malleolus
MPa	Megapascal
PLGA	Poly (lactic-co-glycolic acid)
Ti-6Al-4V	Titanium alloy with 6% Aluminum and 4% Vanadium (α–β alloy)
WE43A	Magnesium alloy containing Yttrium, Rare-Earth Elements, and Zirconium
316LVM	Vacuum-melted low-carbon austenitic stainless steel (implant grade)
MgY4RE3Zr	Magnesium alloy with 4% Yttrium, 3% Rare-Earth Elements, and Zirconium
